# Supplementary figures and images for: Single-cell data revealed CD14-type and FCGR3A-type macrophages and relevant prognostic factors for predicting immunotherapy and prognosis in stomach adenocarcinoma
Source: PeerJ. 2024 Jan 22;12:e16776. doi: 10.7717/peerj.16776 (PMC10809984; doi:10.7717/peerj.16776)

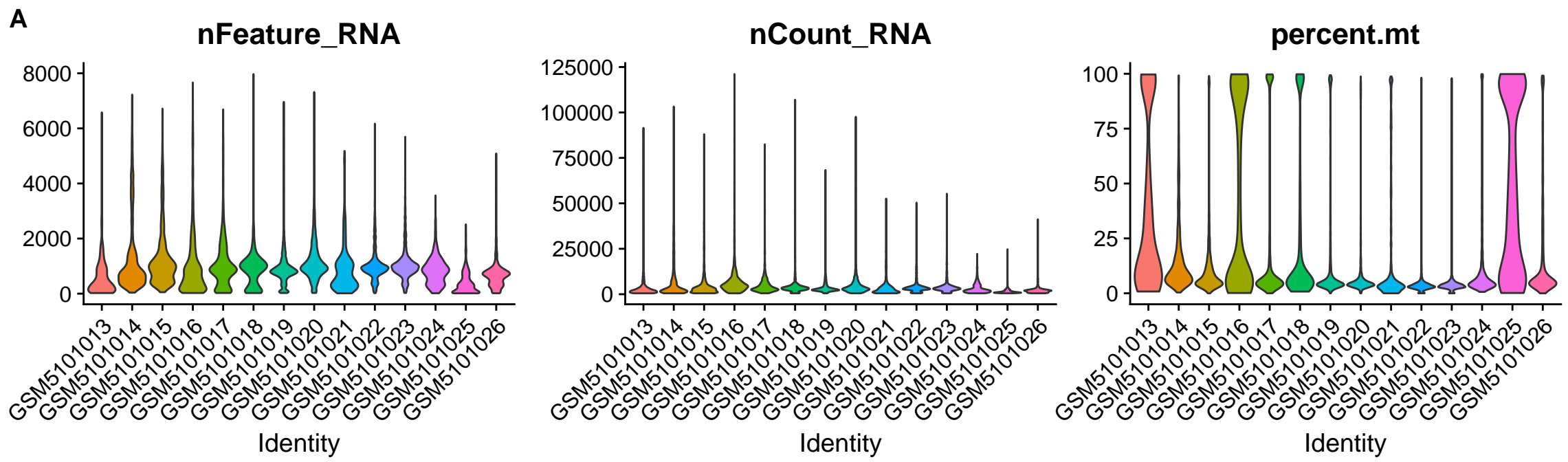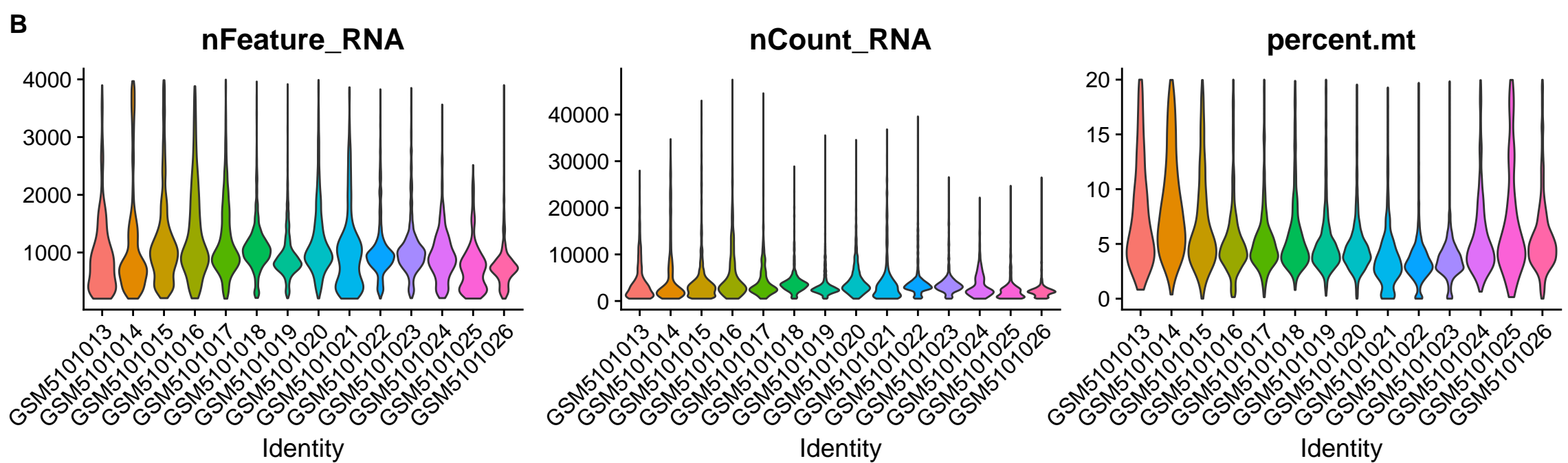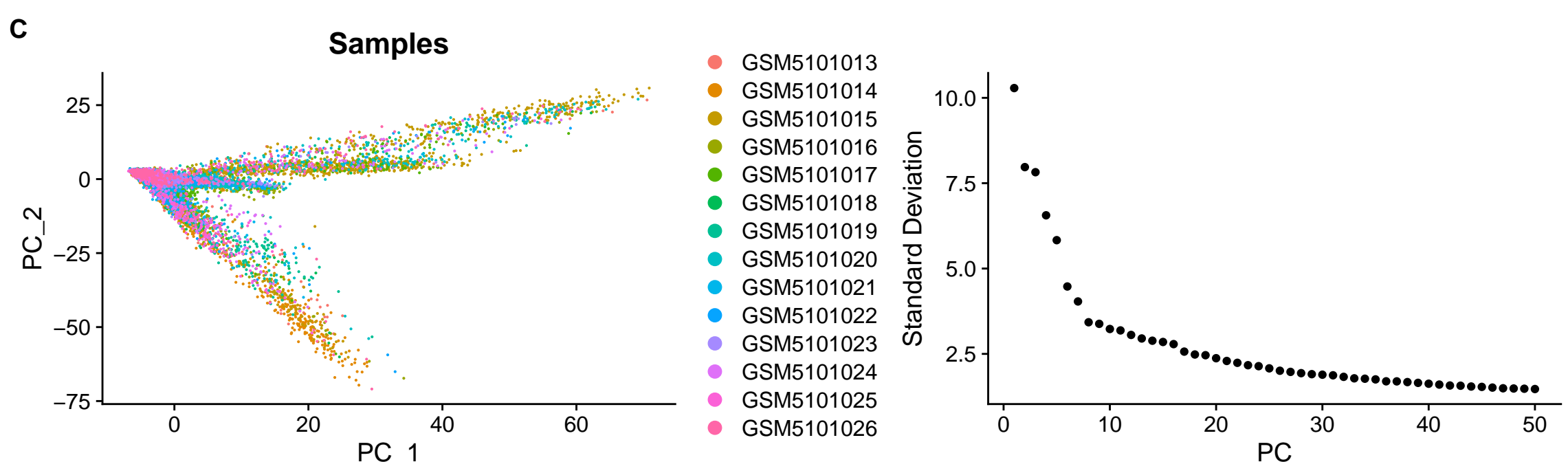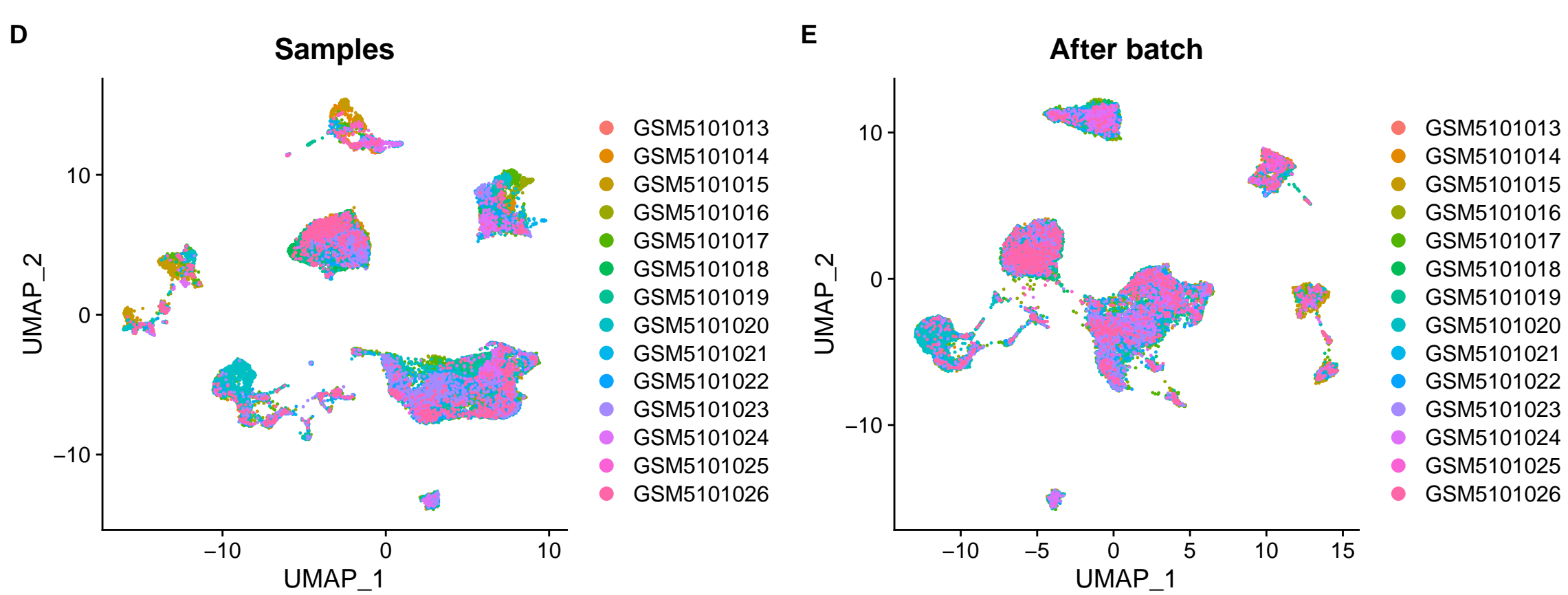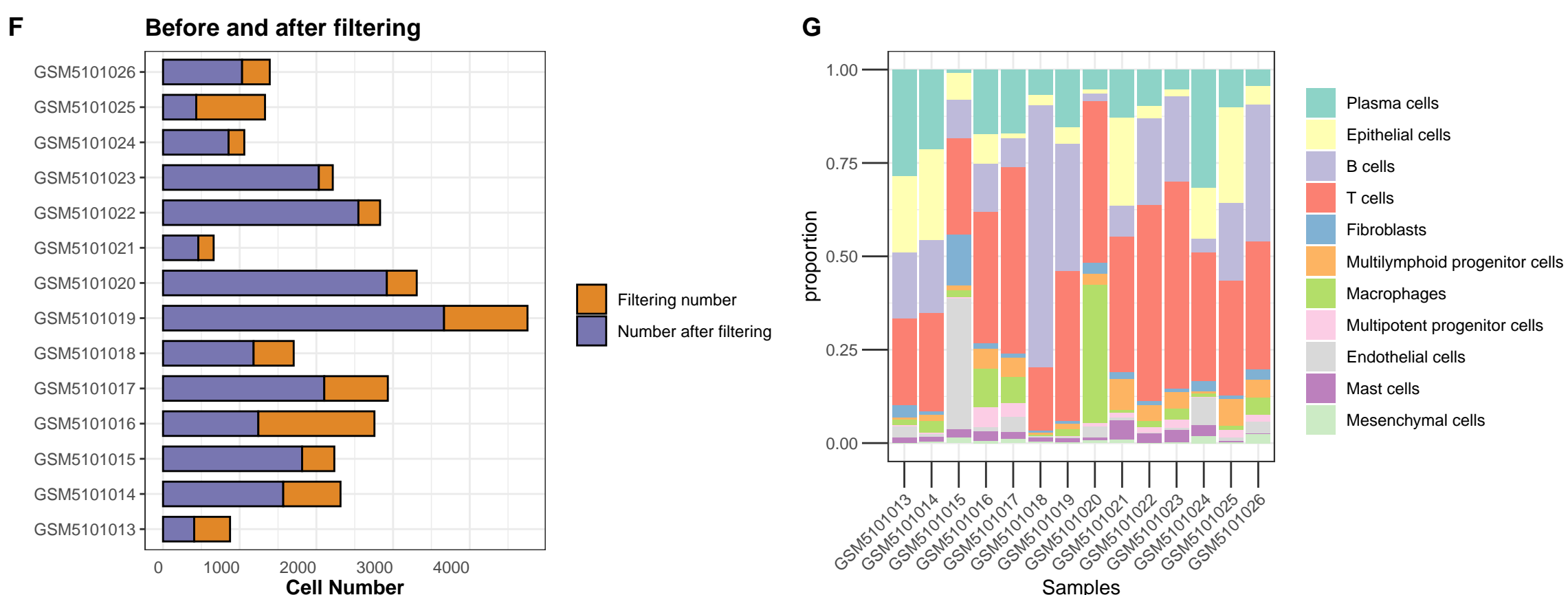

Supplement: Supplemental Information 1 — A: Number of cells, mRNA count and mitochondrial cell ratio before filtering. B: Number of cells, mRNA count and mitochondrial cell ratio after filtering. C: Principal component analysis based on the top 3000 HVGs and the top 50 PCs. D: Cell distribution in samples before removal of batch effect. E: Distribution of cells in samples after removal of batch. F: Number of cells in the sample before and after filtering. G: Proportion of 11 cell types in the sample. [file peerj-12-16776-s001.pdf]

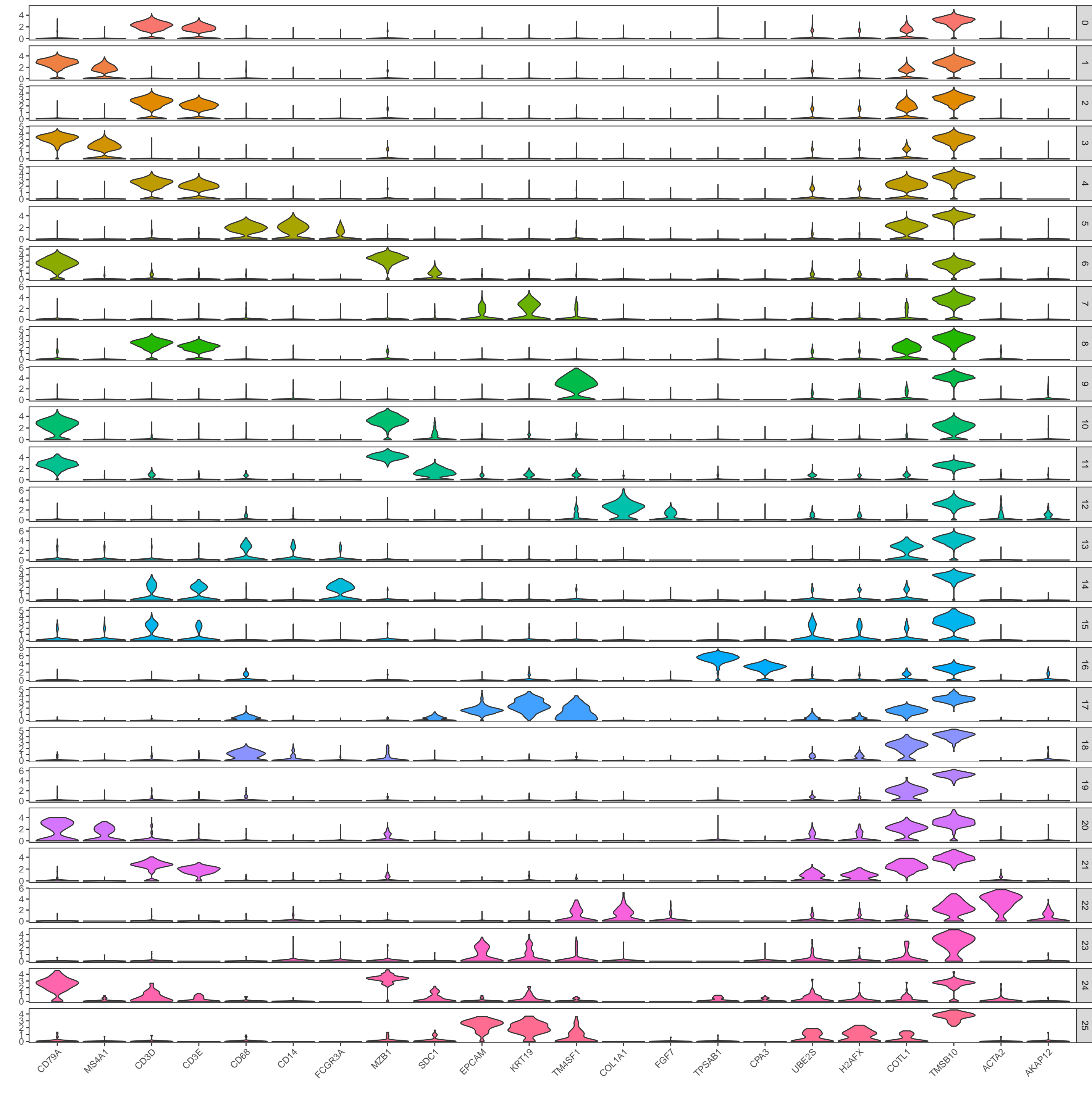

Supplement: Supplemental Information 2 [file peerj-12-16776-s002.pdf]

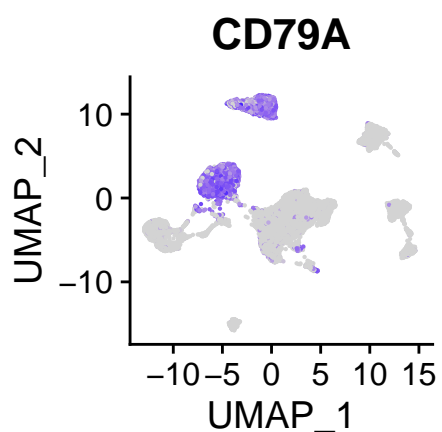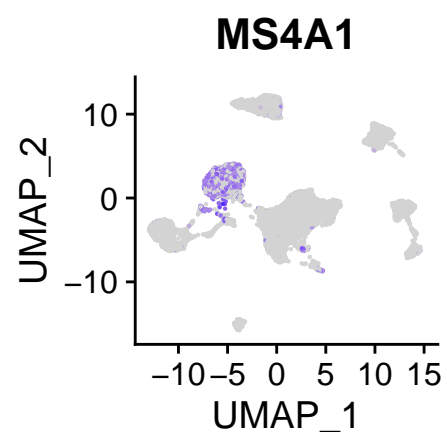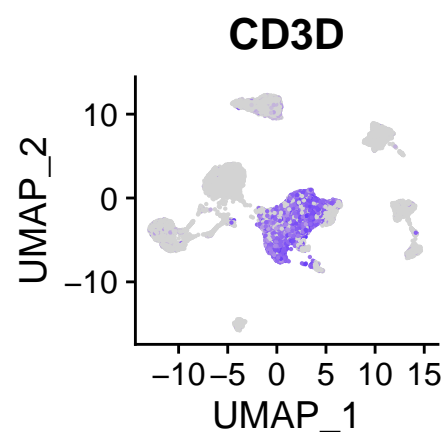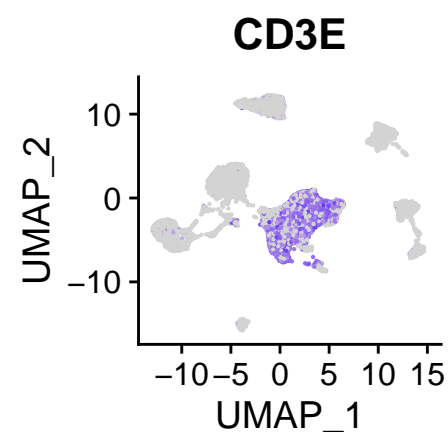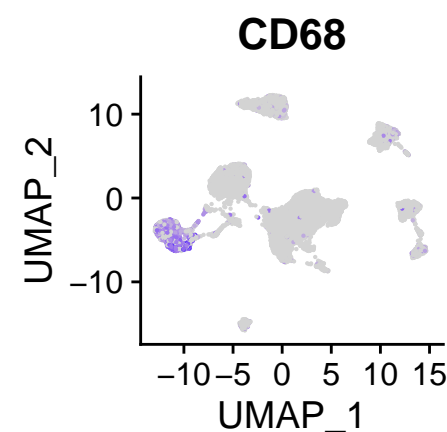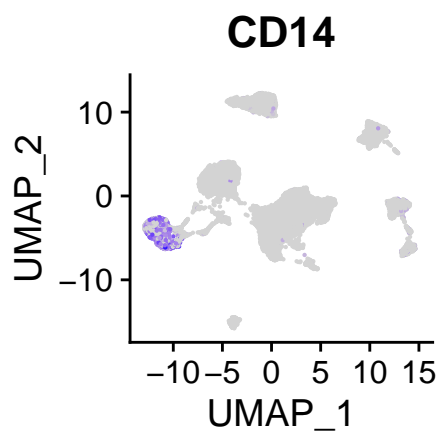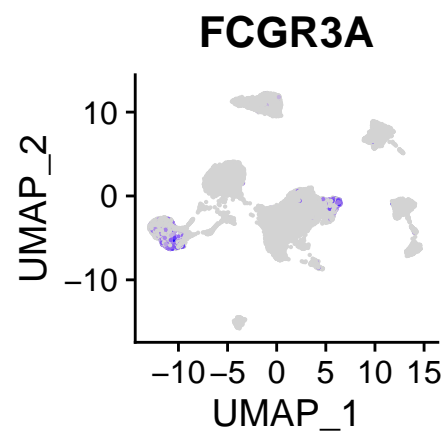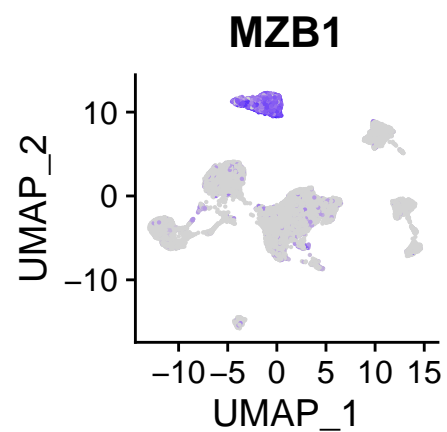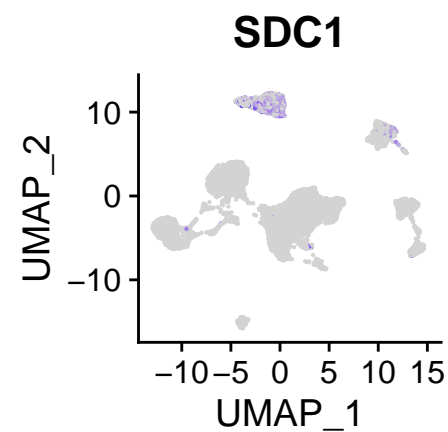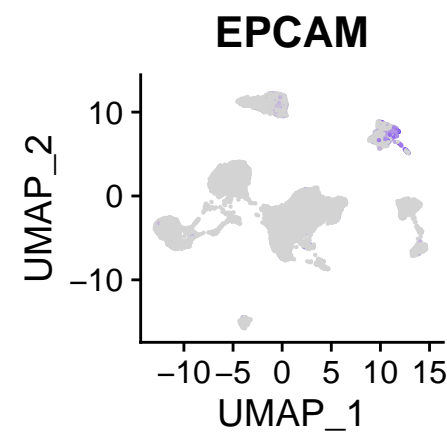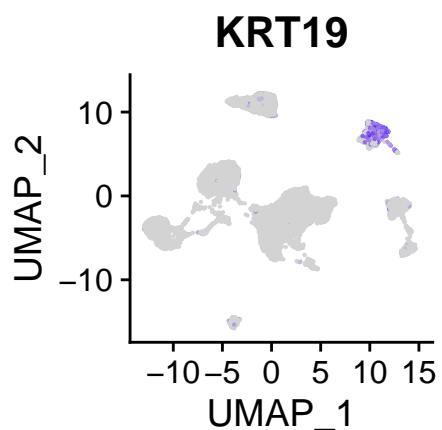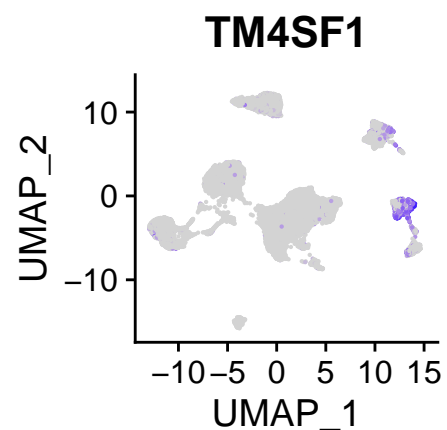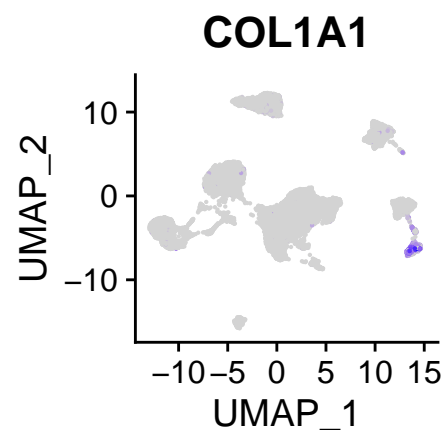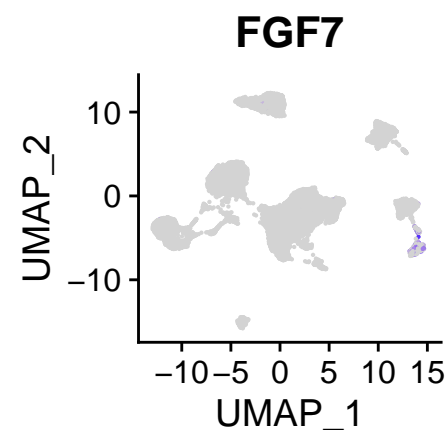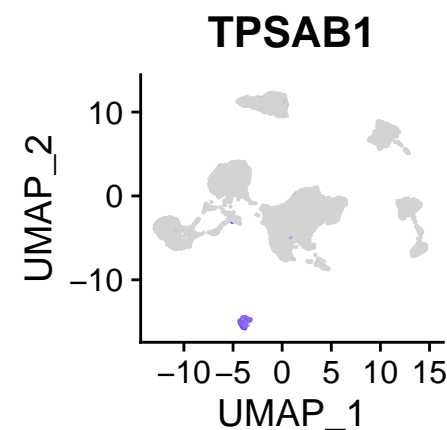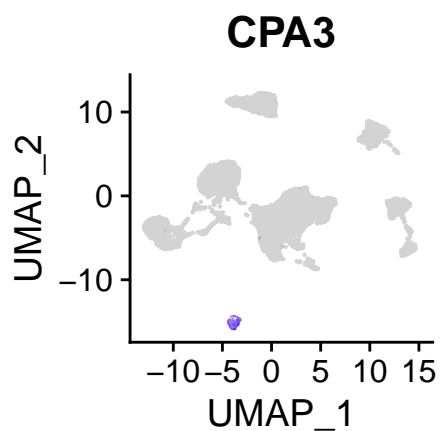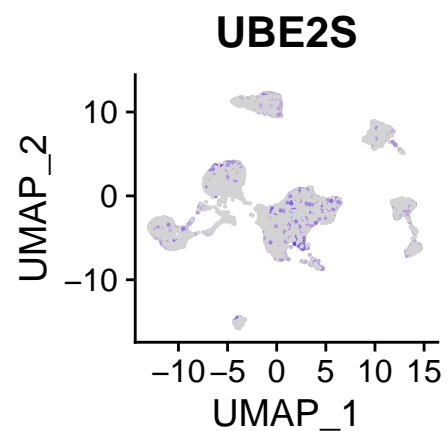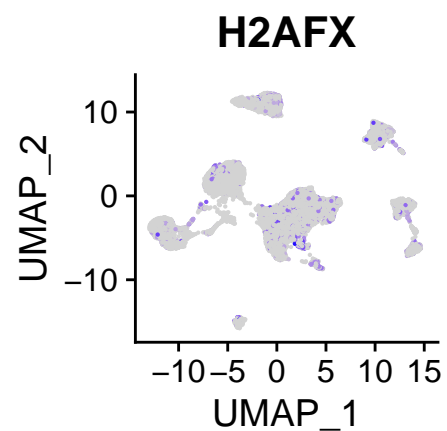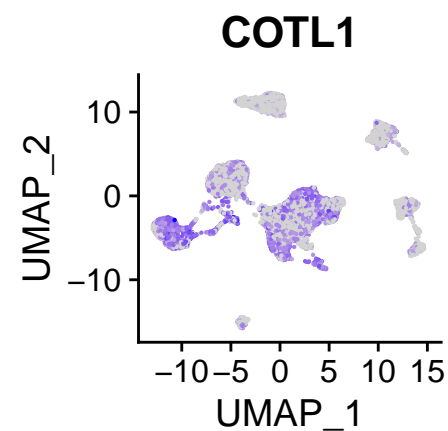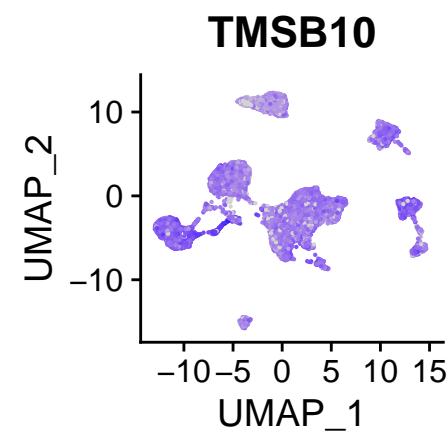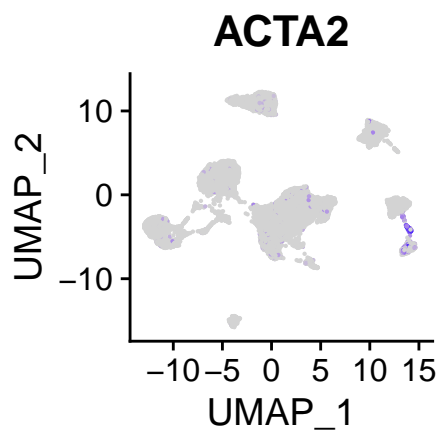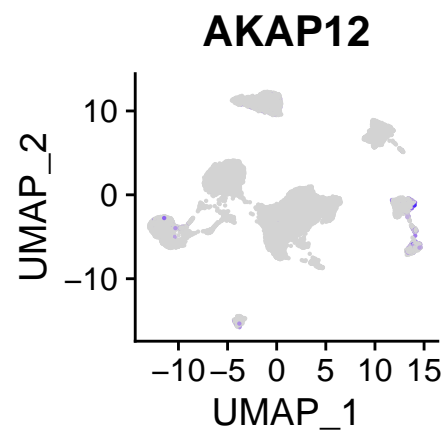

Supplement: Supplemental Information 3 [file peerj-12-16776-s003.pdf]

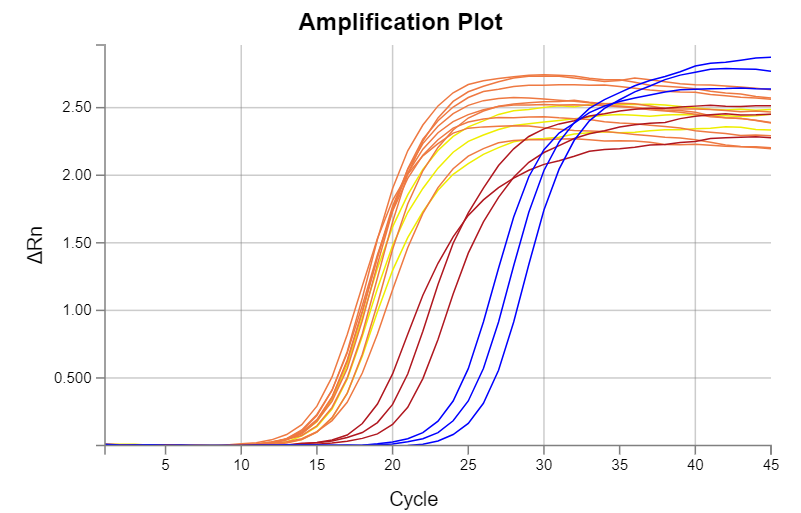

Supplement: Supplemental Information 5 [file peerj-12-16776-s005.zip › Experiment data/PCR/amplification curve/ANXA5.png]

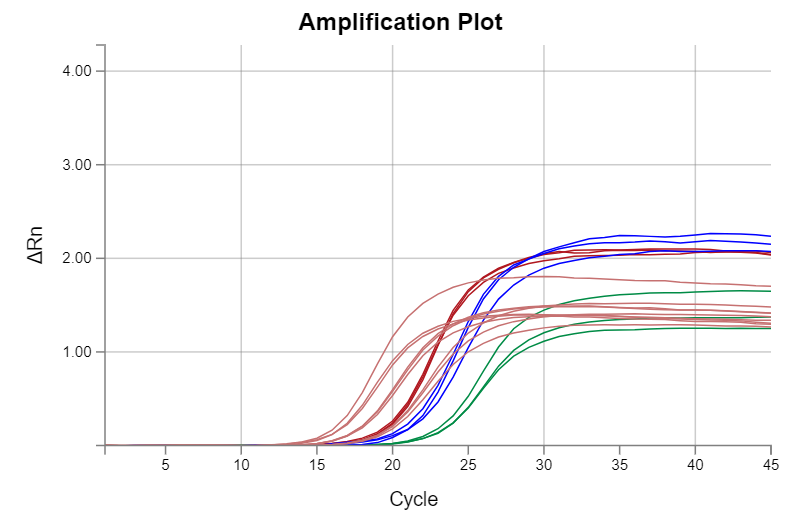

Supplement: Supplemental Information 5 [file peerj-12-16776-s005.zip › Experiment data/PCR/amplification curve/CD36.png]

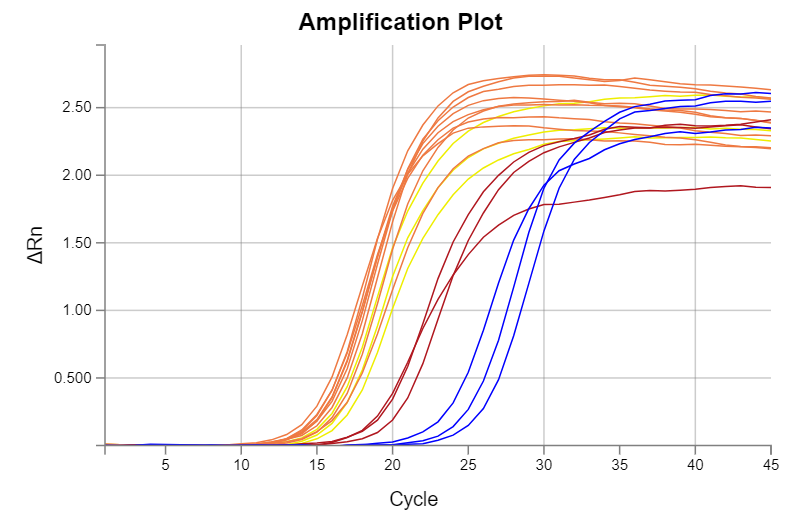

Supplement: Supplemental Information 5 [file peerj-12-16776-s005.zip › Experiment data/PCR/amplification curve/MARCKS.png]

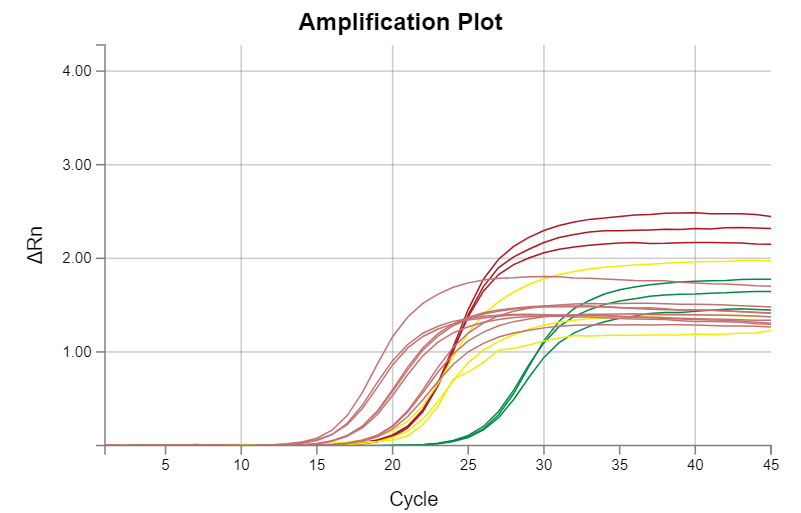

Supplement: Supplemental Information 5 [file peerj-12-16776-s005.zip › Experiment data/PCR/amplification curve/NRP1.png]

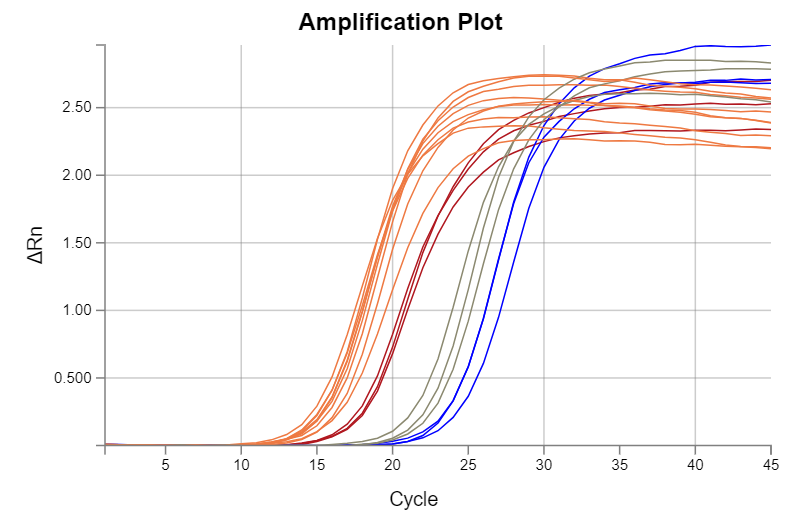

Supplement: Supplemental Information 5 [file peerj-12-16776-s005.zip › Experiment data/PCR/amplification curve/PDE4A.png]

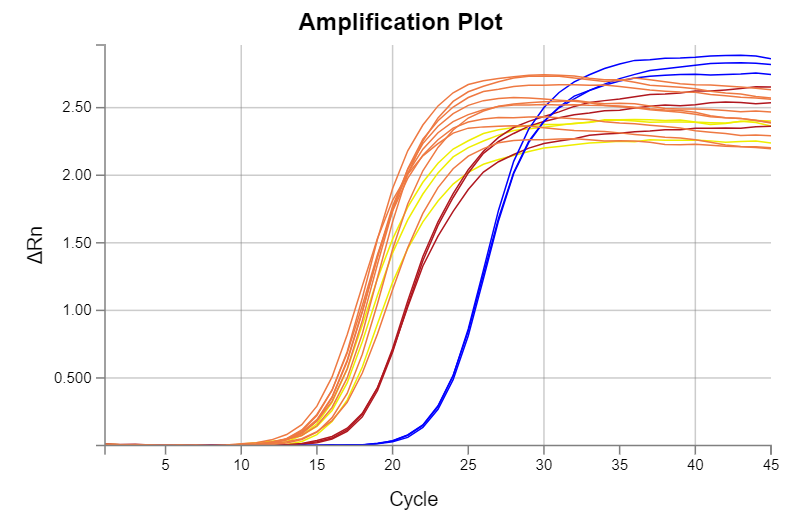

Supplement: Supplemental Information 5 [file peerj-12-16776-s005.zip › Experiment data/PCR/amplification curve/RGS2.png]

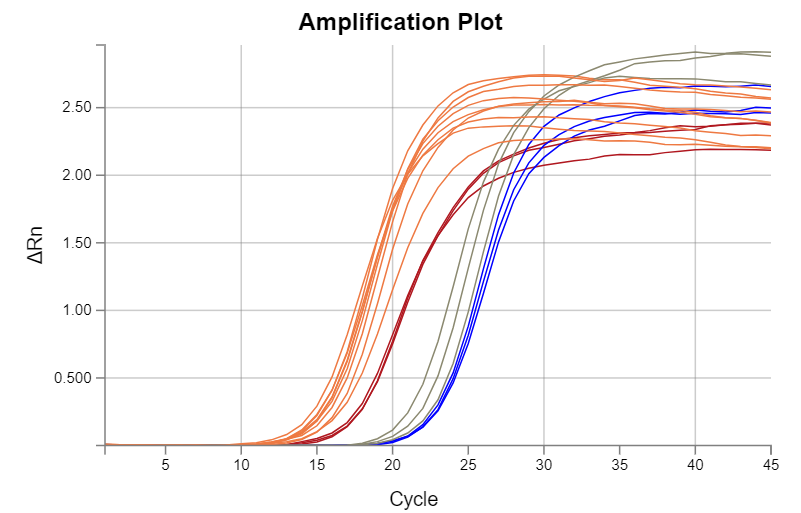

Supplement: Supplemental Information 5 [file peerj-12-16776-s005.zip › Experiment data/PCR/amplification curve/RNAI2.png]

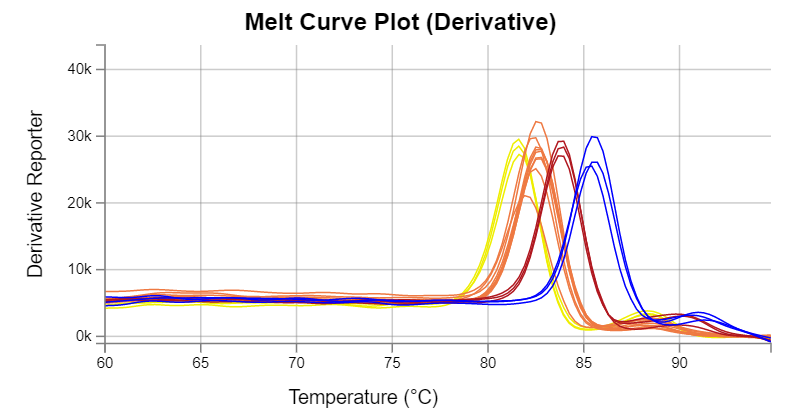

Supplement: Supplemental Information 5 [file peerj-12-16776-s005.zip › Experiment data/PCR/melt curve/Melt ANXA5.png]

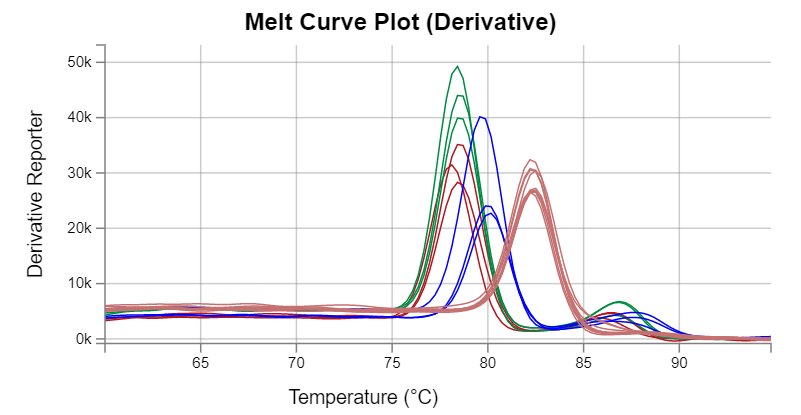

Supplement: Supplemental Information 5 [file peerj-12-16776-s005.zip › Experiment data/PCR/melt curve/Melt CD36.png]

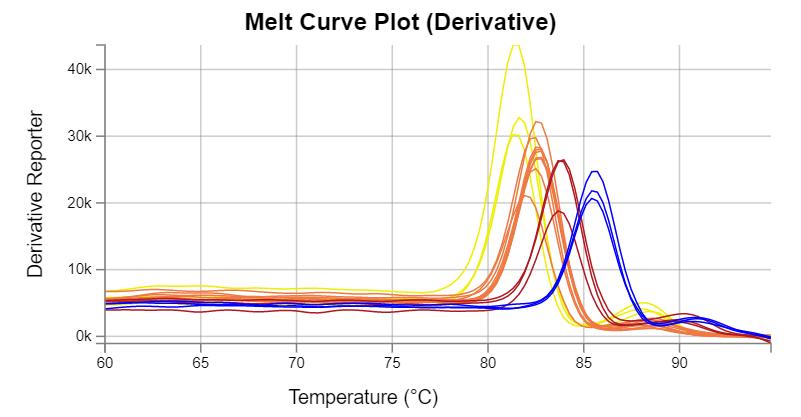

Supplement: Supplemental Information 5 [file peerj-12-16776-s005.zip › Experiment data/PCR/melt curve/Melt MARCKS.png]

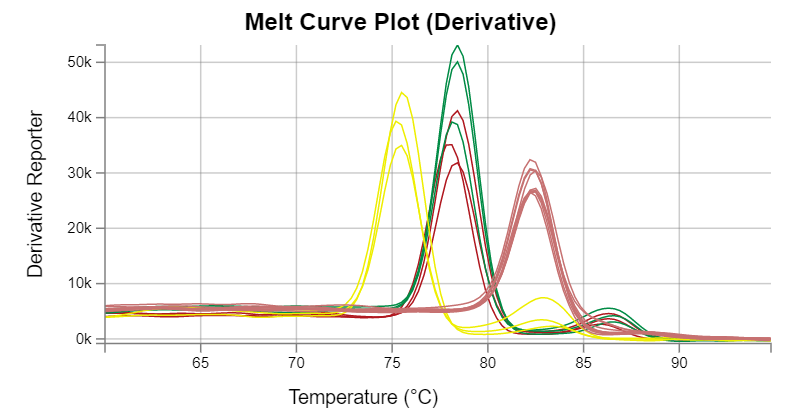

Supplement: Supplemental Information 5 [file peerj-12-16776-s005.zip › Experiment data/PCR/melt curve/Melt NRP1.png]

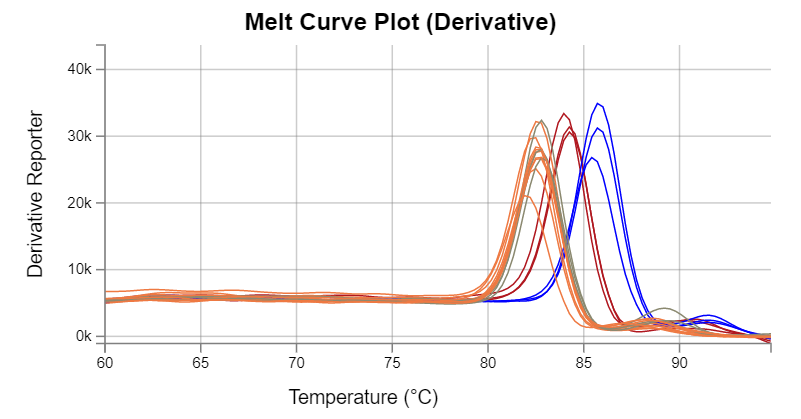

Supplement: Supplemental Information 5 [file peerj-12-16776-s005.zip › Experiment data/PCR/melt curve/Melt PDE4A.png]

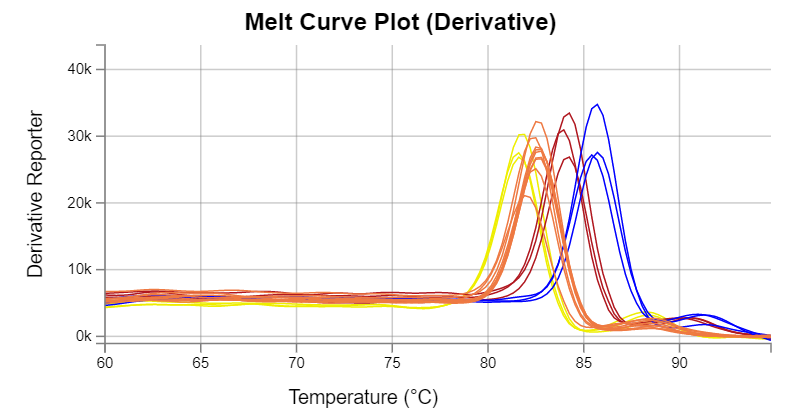

Supplement: Supplemental Information 5 [file peerj-12-16776-s005.zip › Experiment data/PCR/melt curve/Melt RGS2.png]

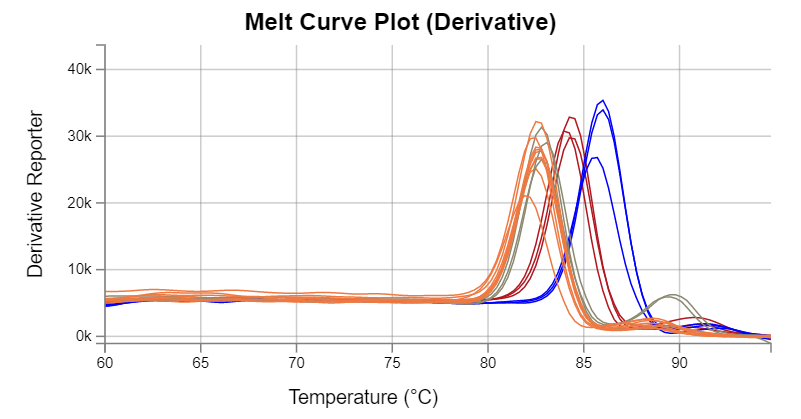

Supplement: Supplemental Information 5 [file peerj-12-16776-s005.zip › Experiment data/PCR/melt curve/Melt RNAI2.png]

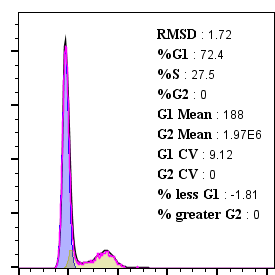

Supplement: Supplemental Information 5 [file peerj-12-16776-s005.zip › Experiment data/cell cycle/MKN28/si MARCK (1).png]

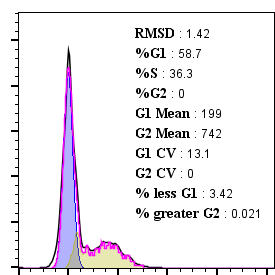

Supplement: Supplemental Information 5 [file peerj-12-16776-s005.zip › Experiment data/cell cycle/MKN28/si MARCK (2).png]

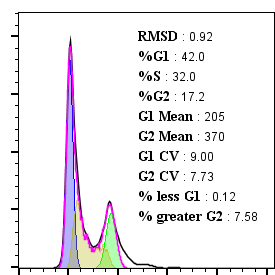

Supplement: Supplemental Information 5 [file peerj-12-16776-s005.zip › Experiment data/cell cycle/MKN28/si MARCK (3).png]

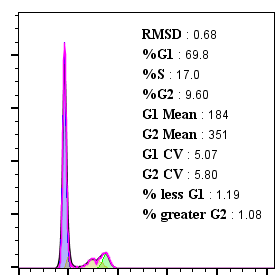

Supplement: Supplemental Information 5 [file peerj-12-16776-s005.zip › Experiment data/cell cycle/MKN28/si NC (1).png]

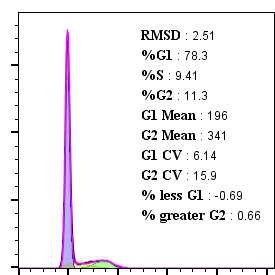

Supplement: Supplemental Information 5 [file peerj-12-16776-s005.zip › Experiment data/cell cycle/MKN28/si NC (2).png]

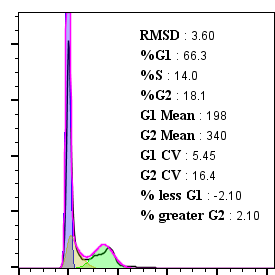

Supplement: Supplemental Information 5 [file peerj-12-16776-s005.zip › Experiment data/cell cycle/MKN28/si NC (3).png]

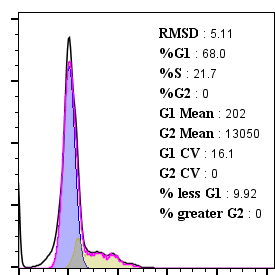

Supplement: Supplemental Information 5 [file peerj-12-16776-s005.zip › Experiment data/cell cycle/MKN7/si MARCK (2).png]

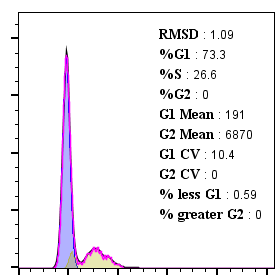

Supplement: Supplemental Information 5 [file peerj-12-16776-s005.zip › Experiment data/cell cycle/MKN7/si MARCK (3).png]

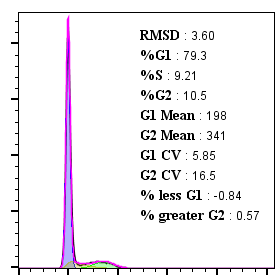

Supplement: Supplemental Information 5 [file peerj-12-16776-s005.zip › Experiment data/cell cycle/MKN7/si NC (1).png]

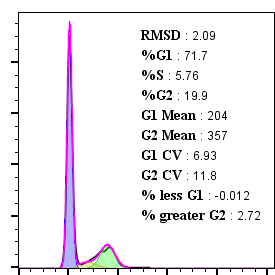

Supplement: Supplemental Information 5 [file peerj-12-16776-s005.zip › Experiment data/cell cycle/MKN7/si NC (2).png]

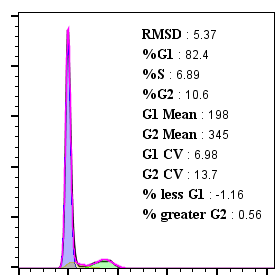

Supplement: Supplemental Information 5 [file peerj-12-16776-s005.zip › Experiment data/cell cycle/MKN7/si NC (3).png]

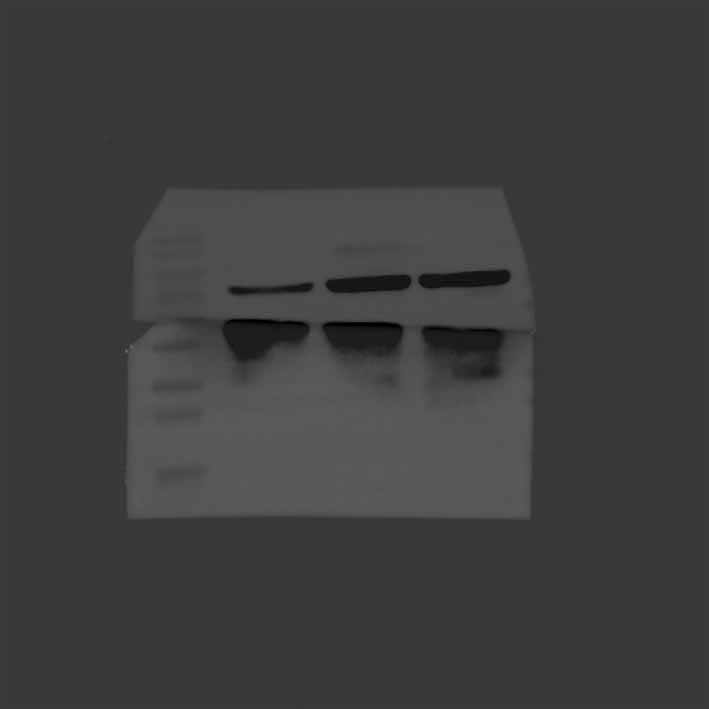

Supplement: Supplemental Information 5 [file peerj-12-16776-s005.zip › Experiment data/wb raw/MARCKS#1.jpg]

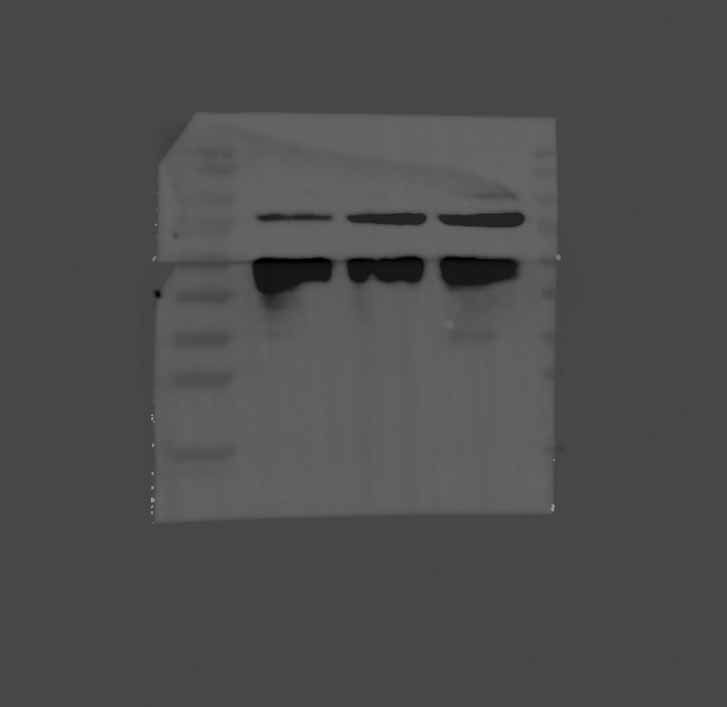

Supplement: Supplemental Information 5 [file peerj-12-16776-s005.zip › Experiment data/wb raw/MARCKS#2.jpg]

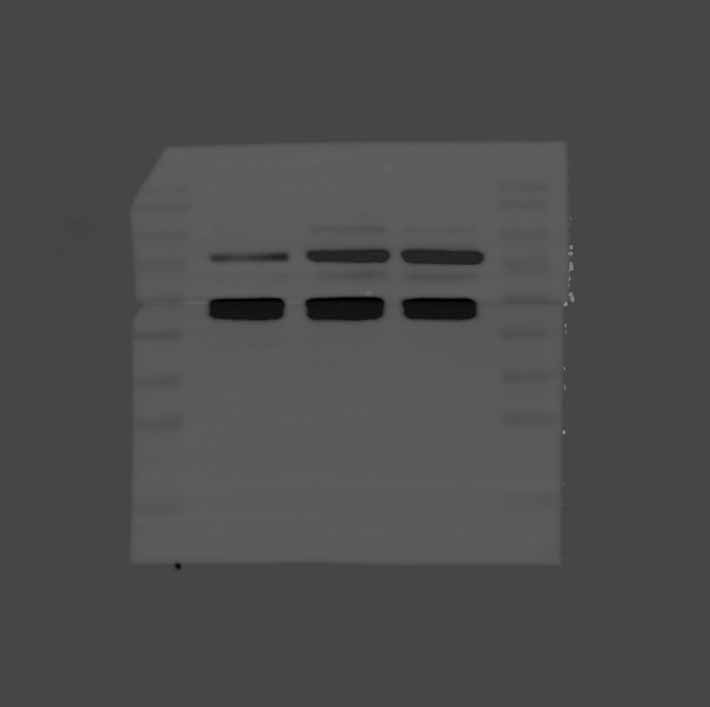

Supplement: Supplemental Information 5 [file peerj-12-16776-s005.zip › Experiment data/wb raw/MARCKS#3.jpg]
