# Supplementary material for: Single-cell data revealed CD14-type and FCGR3A-type macrophages and relevant prognostic factors for predicting immunotherapy and prognosis in stomach adenocarcinoma
Source: PeerJ. 2024 Jan 22;12:e16776. doi: 10.7717/peerj.16776 (PMC10809984; doi:10.7717/peerj.16776)
Supplement: Supplemental Information 4 [file peerj-12-16776-s004.docx]

**MIQE Checklist**

**EXPERIMENTAL DESIGN**

Definition of experimental and control groups

In this experiment, the control group was defined as human normal gastric mucosal epithelial cells (GES-1), while the experimental group was defined as MKN7 and MKN28, the gastric cancer cell lines. These are dealt with in lines 205 to 210 of the manuscript.

Number within each group

The number of each group was 3 independent experiments. These are dealt with in lines 212 to 224 of the manuscript.

**SAMPLE**

Description

Microdissection or microdissection

We did not use techniques such as microdissection or microdissection to obtain samples.

Processing procedure

If frozen - how and how quickly?

We resuspended the MKN7 and MKN28 and GES-1 cell lines using serum-free cell cryopreservative and immediately froze them in a liquid nitrogen tanks. These are dealt with in lines 210 to 211 of the manuscript.

If fixed - with what, how quickly?

No fix.

Sample storage conditions and duration (especially for FFPE samples

Cells were frozen with liquid nitrogen tanks. FFPE samples are not used. These are dealt with in lines 210 to 211 of the manuscript.

**NUCLEIC ACID EXTRACTION**

Procedure and/or instrumentation

Name of kit and details of any modifications

TRIzol reagent (15596026, Thermo Fisher, USA); DEPC water (BL510A, Biosharp, China); chloroform (T819286, MACKLIN, China); isopropanol (I811932, MACKLIN, China); anhydrous ethanol (E809056, MACKLIN, China). These are dealt with in lines 212 to 224 of the manuscript.

Details of DNase or RNAse treatment

The consumables we use are RNAase and DNAase free. Prior to RNA extraction, all consumables were immersed in DEPC water overnight and used after sterilization. These are dealt with in lines 212 to 224 of the manuscript.

Contamination assessment (DNA or RNA)

Contamination assessment was performed by the lab technician. These are dealt with in lines 212 to 224 of the manuscript.

Nucleic acid quantification

Instrument and method

After resuspension of RNA, evaluation of RNA concentration and treatment was performed. We used NanoDrop (ThermoFisher, USA) for RNA concentration and quality assessment. The RNA concentration should be above 500ng/ml and the A260/A280 ratio should be 1.8-2.0. These are dealt with in lines 212 to 224 of the manuscript.

RNA integrity method/instrument

RIN/RQI or Cq of 3' and 5' transcripts

We used agarose gel electrophoresis for RNA integrity experiments. The results showed good RNA integrity. These are dealt with in lines 212 to 224 of the manuscript.

Inhibition testing (Cq dilutions, spike or other)

We did not perform inhibition testing.

**REVERSE TRANSCRIPTION**

Complete reaction conditions

Amount of RNA and reaction volume

We used the High-Capacity cDNA Reverse Transcription Kit (4368814, ThermoFisher, USA) for reverse transcription. RNA volume: 5ul; Reaction volume: 20ul. These are dealt with in lines 212 to 224 of the manuscript.

Priming oligonucleotide (if using GSP) and concentration

The concentration of priming oligonucleotides was 1ul in total 20ul volume. These are dealt with in lines 212 to 224 of the manuscript.

Reverse transcriptase and concentration

The concentration of reverse transcriptase was 1ul in total 20ul volume. These are dealt with in lines 212 to 224 of the manuscript.

Temperature and time

42℃: 60min; 72℃: 5min; 4℃: Save. These are dealt with in lines 212 to 224 of the manuscript.

**qPCR TARGET INFORMATION**

If multiplex, efficiency and LOD of each assay.

No multiplex.

Sequence accession number

RGS2: NM_002923;

RNAI2: NM_001077242;

ANXA5: NM_001154;

MARCK5: NM_002356;

PDE4A: NM_001111307.

CD36: NM_000072.

NRP1: NM_001024628.

These are dealt with in table 1 of the manuscript.

Location of each primer by exon or intron (if applicable)

No applicable.

What splice variants are targeted?

No applicable.

**qPCR OLIGONUCLEOTIDES**

Primer sequences

| Gene | Forward primer sequence (5’-3’) | Reverse primer sequence (5’-3’) |
| --- | --- | --- |
| RGS2 | CTCTACTCCTGGGAAGCCCAAA | TTGCTGGCTAGCAGCTCGTCAA |
| RNAI2 | GTGCCTCCGGCAACATTGA | GCACGAATCTTTGCAGGGA |
| ANXA5 | GTGGCTCTGATGAAACCCTCTC | GGCTCTCAGTTCTTCAGGTGTC |
| MARCK5 | AGCCCGGTAGAGAAGGAGG | TTGGGCGAAGAAGTCGAGGA |
| PDE4A | CTGCGACATCTTCCAGAACCTC | GCTGGTCACTTTCTTGGTCTCC |
| CD36 | CAGGTCAACCTATTGGTCAAGCC | GCCTTCTCATCACCAATGGTCC |
| NRP1 | AACAACGGCTCGGACTGGAAGA | GGTAGATCCTGATGAATCGCGTG |
| GAPDH | AATGGGCAGCCGTTAGGAAA | GCCCAATACGACCAAATCAGAG |

These are dealt with in table 1 of the manuscript.

Location and identity of any modifications

No modifications.

**qPCR PROTOCOL**

Complete reaction conditions

Reaction volume and amount of cDNA/DNA

Reaction volume: 20ul; cDNA amount: 2ul. These are dealt with in lines 212 to 224 of the manuscript.

Primer, (probe), Mg++ and dNTP concentrations

Sense primer: 0.5ul; antisense primer: 0.5ul; Mg++ concentration: 25nm/ml; dNTP concentrations: 100umol/l. These are dealt with in lines 212 to 224 of the manuscript.

Polymerase identity and concentration

Polymerase identity: RevertAidRNAse Reverse Ensyme (200);

Polymerase concentration: 1ul. These are dealt with in lines 212 to 224 of the manuscript.

Temperature and time

Buffer/kit identity and manufacturer: High-Capacity cDNA Reverse Transcription Kit (4368814, ThermoFisher, USA). These are dealt with in lines 212 to 224 of the manuscript.

Additives (SYBR Green I, DMSO, etc.)

FastStart Universal SYBR ®Green Master (Roche). These are dealt with in lines 212 to 224 of the manuscript.

Complete thermocycling parameters

Cycling conditions started with an initial DNA denaturation phase at 95°C for 30 seconds, followed by 45 cycles at 94°C for 15 seconds, 56°C for 30 seconds, and 72°C for 20 seconds. These are dealt with in lines 212 to 224 of the manuscript.

Manufacturer of qPCR instrument

LightCycler 480 PCR System (Roche). These are dealt with in lines 212 to 224 of the manuscript.

**qPCR VALIDATION**

Specificity (gel, sequence, melt, or digest)


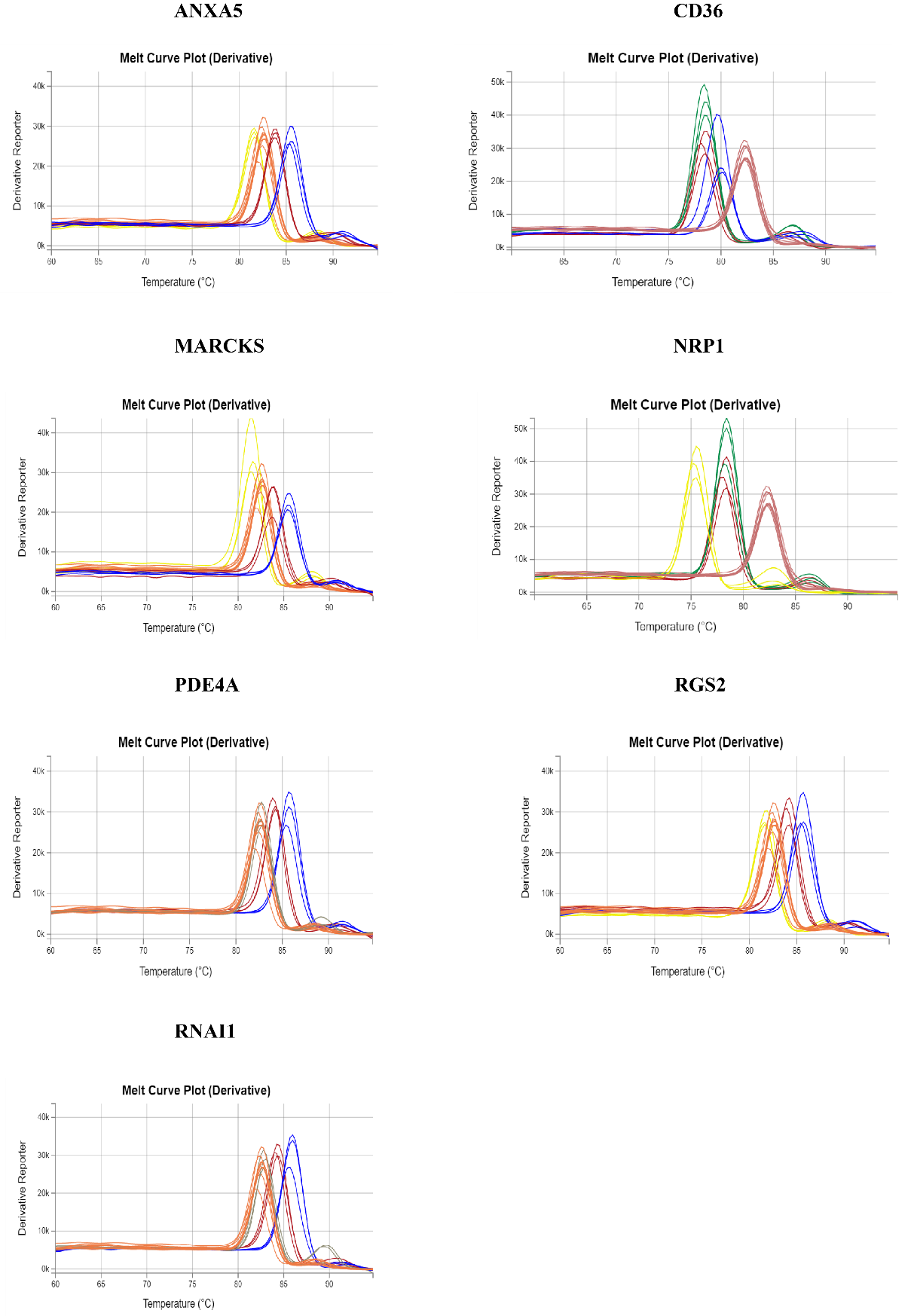


For SYBR Green I, Cq of the NTC

Cq: 44 cycle. These are dealt with in raw data, and we uploaded it.

Standard curves with slope and y-intercept

PCR efficiency calculated from slope

No calculation.

R^2 of standard curve

R^2=0.99. These are dealt with in raw data.

Linear dynamic range

Linear dynamic range is in the acceptable range.

Cq variation at lower limit

10 bp. These are dealt with in raw data.

Evidence for limit of detection

A relatively quantitative comparative CT method was used.

If multiplex, efficiency and LOD of each assay.

Not multiplex.

**DATA ANALYSIS**

qPCR analysis program (source, version)

ThermoFisher Design and analysis software 2.6.0. These are dealt with in lines 212 to 224 of the manuscript.

Cq method determination

We take the start of the amplification curve as the Cq value. These are dealt with in lines 212 to 224 of the manuscript.

Outlier identification and disposition

We remove outliers.

Results of NTCs

We found no abnormalities in the NTCs.

Justification of number and choice of reference genes

The reference gene is GAPDH, which is the reference gene chosen in most studies. These are dealt with in lines 212 to 224 of the manuscript.

Description of normalisation method.

We normalized the results by GAPDH. The Cq value of the corresponding GAPDH was subtracted from the Cq value of the sample set. These are dealt with in lines 212 to 224 of the manuscript.

Number and concordance of biological replicates

Three separate analyses were performed on each sample. These are dealt with in lines 212 to 224 of the manuscript.

Repeatability (intra-assay variation)

Repeatability is fine.

Statistical methods for result significance

Statistical comparisons between two and multiple groups were respectively performed using Student's t-test and one-way analysis of variance followed by either a Dunnett’s test. These are dealt with in lines 237 to 245 of the manuscript.

Software (source, version)

Excel and Graphpad Prism 9.4.1. These are dealt with in lines 237 to 245 of the manuscript.
